# The effect of an 8-week treatment program using a novel foot neuromuscular electrical stimulator on physical function, leg pain, leg symptoms, and leg blood flow in community-dwelling older adults: a randomized sham-controlled trial

**DOI:** 10.1186/s13063-022-06828-2

**Published:** 2022-10-14

**Authors:** Binoy Kumaran, Darren Targett, Tim Watson

**Affiliations:** 1 School of Health and Social Work, University of Hertfordshire, Hatfield, UK; 2 Primoris Contract Solutions Ltd, 22 Redwood Drive, Ascot, UK

**Keywords:** Neuromuscular electrical stimulator (NMES), Blood flow, Leg pain, Leg symptoms

## Abstract

**Background:**

Neuromuscular electrical stimulation (NMES) is an established therapy that has been widely used for many decades to improve circulation in the legs. However, studies using NMES devices in an elderly, ambulant, and otherwise apparently healthy population are lacking; this is despite the use of such devices being indicated for lower leg symptoms (such as aches, pain, and cramps) that are frequently seen in older individuals. The main purpose of this study is to evaluate the effect of non-invasive foot NMES (administered using Revitive Medic^©^) on such symptoms compared to a sham in a 12-week period.

**Methods:**

This is a single-center, single (participant)-blind, parallel-group, randomized, placebo-controlled (sham group), interventional study. Participants will be randomized to 1 of 3 groups (1:1:1) with each study group receiving a different type of foot NMES: Revitive sham; Revitive Medic^©^ Program 1; or Revitive^®^ Program 2. Each participant will be instructed to self-administer the foot NMES device for 30 min twice daily for 8 weeks. During the study, all participants will continue with their normal life, activities, medications, and diet with no restrictions. Following the 8-week NMES treatment program participants will be assessed for Canadian Occupational Performance Measure performance (COPM-P) and satisfaction (COPM-S) scores, lower leg pain, lower leg symptoms (heaviness, tiredness, aching and cramps), and blood flow volume and intensity.

**Discussion:**

Revitive^®^ foot NMES has been proven to increase blood circulation in the legs during use, which may help to relieve symptoms such as pain, heaviness, cramps, and tiredness. When NMES is applied to the plantar surface of the feet it indirectly induces contraction of the calf muscle, activating the musculo-venous pump and thus improving circulation. This study aims to provide data informing on the applicability of foot NMES for the management of leg symptoms that are likely to be indicative of poor circulation in an elderly (> 65 years) community population.

**Trial registration:**

ISRCTN10576209.

## Administrative information

Note: the numbers in curly brackets in this protocol refer to SPIRIT checklist item numbers. The order of the items has been modified to group similar items (see http://www.equator-network.org/reporting-guidelines/spirit-2013-statement-defining-standard-protocol-items-for-clinical-trials/).

**Table:** 

Title {1}	The effect of an 8-week treatment program using a novel foot neuromuscular electrical stimulator on physical function, leg pain, leg symptoms and leg blood flow in community-dwelling older adults: a randomized sham-controlled trial]
Trial registration {2a and 2b}.	ISRCTN10576209
Protocol version {3}	Version 4.0, dated 12 July 2022.
Funding {4}	Actegy Limited (Bracknell, UK) provided funding for: development of the trial protocol and analysis plan; carrying out all aspects of the trial including participant recruitment, outcome assessment, delivery of the intervention, monitoring data quality, data analysis and reporting, and publication of findings; fixed costs including printing, equipment and software licensing; participant-related costs including consumables, recruitment-related costs, and participant transport; staff costs for the trial coordinator and research assistants, and consultancy costs for statistical and medical support as required. Statistical analysis is also funded by Actegy. Actegy Limited provides Revitive and sham devices for use in the trial.
Author details {5a}	B Kumaran, School of Health and Social Work, University of Hertfordshire, Hatfield, UK. D Targett, Primoris Contract Solutions Ltd, 22 Redwood Drive, Ascot, UK T Watson, School of Health and Social Work, University of Hertfordshire, Hatfield, UK.
Name and contact information for the trial sponsor {5b}	University of Hertfordshire, Hatfield, UK
Role of sponsor {5c}	The University of Hertfordshire takes responsibility for the trial protocol, conduct and reporting.

## Introduction

### Background and rationale {6a}

Lower leg symptoms such as aching/pain, leg heaviness, tiredness, and cramping are common in older adults and impact considerably on physical functioning and quality of life. A consequential deterioration in physical functioning due to lower leg symptoms is often considered part of the normal aging process [1]. Functional limitation is multifactorial in origin but, in part, reflects self-restriction of activity to reduce the occurrence of leg discomfort [2]. However, these lower leg symptoms can develop due to prolonged inactivity, a sedentary lifestyle, and restricted movement/activity due to medical circumstances. Associations between pain, low levels of physical activity, and physical limitation have led to the development of a “vicious cycle” model in which leg symptoms limit physical activity; in turn limitation of activity levels increases the severity of leg symptoms [3]. Such symptoms can indicate poor blood flow to the legs [1, 4–6].

Peripheral artery disease is a condition that occurs because of the development of atherosclerotic lesions that limit blood flow to the legs. Reduced blood flow because of peripheral artery disease is associated with a number of physiological manifestations in the lower extremities including vascular dysfunction, altered muscle metabolism, impaired angiogenesis, and inflammatory activation all of which contribute to symptoms of limb discomfort and functional limitation [2]. It is thought that spending prolonged periods of time in a static or sedentary position not only contributes to reduced blood flow but also exacerbates the development of atherosclerotic lesions, as blood flow exerts shear stress across the endothelial surface [7].

The traditional symptom of peripheral artery disease is intermittent claudication, whereby limited flow of oxygenated blood to the legs causes pain on walking that resolves with rest [8]; definitive diagnosis however requires demonstration of low ankle brachial index [1]. Most patients with peripheral artery disease do not present with intermittent claudication: 20% to 50% are reported with no exertional leg symptoms; and 40% to 50% report atypical leg symptoms such as pain/discomfort at rest, pain/discomfort that does not cause them to stop walking, or pain/discomfort that does not resolve with rest [1]. Some individuals with peripheral artery disease report no exertional leg symptoms but may restrict their physical activity as an avoidance mechanism.

It is widely understood that peripheral artery disease is underdiagnosed, in part because of the variety of leg symptoms associated with the condition but also because asymptomatic disease occurs in up to 60% of patients [9]. The prevalence and significance of low normal and abnormal ankle brachial index in a community-dwelling population of sedentary, older individuals is largely unknown. In the LIFE study which considered more than 1566 community-dwelling, sedentary individuals aged 70 to 89 years, 14% of participants were identified as having peripheral artery disease based on ankle brachial index; 16% had borderline peripheral artery disease, 33% had low normal ankle brachial index, and 37% had no peripheral artery disease. This highlights that peripheral artery disease is common among the community-dwelling population of sedentary-older population but is often undiagnosed. Low ankle brachial index was associated with older age and poorer mobility in terms of 400-m walk time and 4-m walk velocity [10].

NMES is an established therapy that has been widely used for many decades as a treatment for muscle impairment associated with various musculoskeletal, neurological, and vascular conditions [11]. It involves the repeat application of a low-level electrical current to facilitate muscle contraction. NMES programs are typically composed of a sequence of stimulation-rest cycles across a range of frequencies and intensities to generate repeated muscle contraction [12]. When NMES is applied to the plantar surface of the feet it indirectly induces contraction of the calf muscle which: activates the musculo-venous pump, increases arterial and venous flow and microcirculation, reduces venous stasis, increases interstitial hydrostatic pressure, promotes interstitial tissue fluid reabsorption, increases lymphatic flow and improves muscle tone [13–19]. Improved circulation delivers more oxygenated blood to the legs, feet, and ankles and helps maintain leg circulatory health, while regular muscle stimulation increases muscle strength to help improve mobility.

Foot NMES devices have been demonstrated to boost blood circulation [20–22] in the legs thereby relieving symptoms such as aching/pain, heaviness, tiredness, and cramps. In healthy individuals, NMES application to the feet using the Revitive footplate significantly increased median lower limb arterial and venous blood flow during the procedure [23]. Subsequent studies of NMES using the Revitive footplate in individuals with peripheral artery disease showed significant increases in blood volume flow and time-adjusted mean blood velocity during device use; this was associated with amelioration of claudication symptoms during exercise, increased exercise capacity, and improved disease-specific quality of life [24]. Similarly, studies in patients with chronic venous disease demonstrate that NMES significantly increases venous flow parameters and ameliorates the effect of orthostasis on fluid accumulation during use [25, 26].

This study aims to investigate the effects of non-invasive NMES delivered via a footplate device (Revitive Medic^©^) on lower leg blood flow and symptoms in community-dwelling adults over the age of 65 years.

The study will consider the effects of NMES delivered by two Revitive Medic^©^ programs in comparison with sham treatment; Revitive Medic^©^ Program 1 uses an NMES program of 15 waveforms that runs twice over 30 min to stimulate blood flow in the feet and lower legs; Revitive^®^ Program 2 comprises 6 waveforms delivered in a package of 10 that is run 3 times over 30 min.

### Objectives {7}

The primary objective of the study is to evaluate the efficacy of non-invasive foot NMES administered using Revitive Medic^©^ Program 1 over 8 weeks compared with Revitive sham. The secondary objective is to evaluate the efficacy of non-invasive foot NMES administered using Revitive^®^ Program 2 over 8 weeks compared with Revitive sham.

### Trial design {8}

This is a single-center, single (participant)-blind, parallel-group, randomized, placebo-controlled (sham group), interventional study. Participants will be randomized to 1 of 3 groups (1:1:1) with each study group receiving a different type of NMES:

- Group 1—Revitive sham
- Group 2—NMES using Revitive Medic^©^ Program 1
- Group 3—NMES using Revitive^®^ Program 2.

Each participant will be instructed to self-administer the foot NMES device for 30 min twice daily over 8 weeks. Treatment will be administered in a seated position with the participant placing the soles of their feet onto the rubberized foot plates. The device is timed to run continuously for 30 min.

A hierarchical testing approach will be used with comparison of Revitive sham versus Revitive Medic^©^ Program 1 as the primary endpoint; if a statistically significant difference is seen for the primary endpoint, Revitive sham versus Revitive^®^ Program 2 will be tested as a secondary endpoint. The study will investigate if Revitive Medic Program 1 is “superior” compared to Sham, and if that is true, then will go on to investigate if Revitive Program 2 is ‘superior’ compared to Sham.

## Methods: participants, interventions, and outcomes

### Study setting {9}

The study will be conducted at the School of Health and Social Work, University of Hertfordshire, Hatfield, UK.

This is a community study in which participants self-administer their randomized NMES device at home. The Revitive Medic^©^ is intended for use in the home setting without the need for supervision by a clinical professional; the device is widely available for purchase, without requiring a prescription. The device is simple to use with minimal instruction. Participants in the trial will be familiarized with the device at the initial study visit and can contact the investigator to address any queries or concerns.

### Eligibility criteria {10}

Eligible participants will be community-dwelling adults aged > 65 years who have one or more of the following symptoms in one or both legs: aching/pain in the leg, heaviness in the leg, tiredness in the leg, or cramps in the leg at any time in the day or night.

Exclusion criteria will comprise (but not limited to) severe diabetes mellitus with severe diabetic neuropathy; lumbar radiculopathy; restless legs syndrome; active cancer; standard contraindications to NMES treatment such as the presence of an electronic implanted device (e.g., cardiac pacemaker); any significant injury to the leg(s) in the last 6 months; being non-ambulant; inability to communicate in English; and inability to provide informed consent.

### Who will take informed consent? {26a}

Potential study participants will initially be contacted by telephone, at which time the nature of the study will be discussed. It will be explained that full participation in the study depends upon whether they meet eligibility criteria, and participant eligibility for the study will be reviewed. Eligible and willing participants will be entered into the study during the telephone call. After the call participants will be randomized and sent the participant information sheet to review prior to their first study visit. At the first visit, the investigator will review the participant information sheet with the participant and ask if they understand the purpose of and their involvement in the study. Prior to any screening assessments, participants will be asked to voluntarily provide informed consent to participate in the study; if the participant is unsure of any aspect of the study, time will be taken to clarify study details. It will also be explained that the participant can decline the option to participate or can withdraw from the study at any time without needing to give a reason.

### Additional consent provisions for collection and use of participant data and biological specimens {26b}

N/A—participant data will not be used in ancillary studies.

## Interventions

### Explanation for the choice of comparators {6b}

The comparator chosen for the study is “Revitive sham.” This will enable the study to demonstrate how much placebo effect, if any, is achieved with the use of a sham treatment. The comparison between the Revitive sham and the intervention(s) will demonstrate whether the real treatment effects achieved with the active devices are statistically and/or clinically significantly different (i.e., superior) to the placebo effect. To enable successful blinding of the participants, the appearance and all functionalities of the sham device are kept identical to the active devices; however, unlike in the active devices, the stimulation intensity in the Revitive sham is limited to a maximum of 2 (of 99) units. Limiting the intensity level to 2 ensures that a clinically meaningful NMES is not delivered to the tissues, and it is assumed that any potential clinical benefit that may be achieved from this sham application amounts to a placebo effect.

### Intervention description {11a}

#### Revitive Medic© Program 1 and Revitive^®^ Program 2

Revitive, is an approved, commercially available, Class IIa NMES medical device produced by Actegy Limited that stimulates the sensory and motor nerves of the feet and lower legs; the device is CE marked and indicated to manage leg pain and discomfort and/or poor circulation due to inactivity, osteoarthritis, diabetes, peripheral arterial disease, and chronic venous insufficiency. Revitive Medic^©^ Program 1 uses device 3156ADi and a modified device (5520AR) is used for Revitive^®^ Program 2. The difference between these two devices is in the waveform design and the number and order of waveforms. Revitive Medic^©^ Program 1 uses a program of 15 waveforms that runs twice over 30 min; Revitive^®^ Program 2 comprises 6 waveforms delivered in a package of 10 that is run 3 times over 30 min. The electrical stimulation intensity is variable and is controlled and set by the user from 1 to 99 units.

Both devices deliver intermittent, low-frequency impulses through surface electrodes on the footpad to produce (strong) skeletal muscle contraction because of intramuscular nerve branch activation. The devices have an “IsoRocker,” which allows it to gently rock back and forth, creating involuntary ankle movement to replicate heel-toe raises. Both devices are pre-programmed to deliver a series of stimulation patterns that last approximately 60 s each; each pattern is made up of a sequence of stimulation pulses delivered in cycles at frequencies of 20 to 44 Hz and durations of 450–970 μs. Therapeutic levels are dependent on reaching an intensity sufficient to induce motor neurostimulation that causes calf muscle contraction but remains comfortable for the user. The optimal intensity level to induce muscle contraction varies from person to person and may depend on factors including plantar surface skin hydration and comfort.

When first using their device at the baseline assessment visit, baseline sensory and motor thresholds will be established by systematically increasing stimulation intensity in increments of one unit, while the participant provides verbal feedback. The stimulation intensity will be adjusted to the higher limit of what the participant can tolerate; participants will be instructed to use the device at the optimal level identified at their study visit and increase it as necessary to maintain a level that is strong but comfortable for them. Participants will be advised to complete two 30-min sessions each day over the 8-week study period, with sessions being roughly 12 h apart.

#### Revitive sham

Device 3714AN will be used for sham treatment. The only difference between the sham and active devices is in the intensity setting. As explained above, the maximum intensity that a Revitive sham device can deliver is capped at 2 (unlike 99 in the active devices) although the intensity display on the sham device is variable reading up to 99, ensuring that the display is the same as for the active devices. If a participant increases the intensity on the control pad, they will see an increase on the display, but the actual intensity is capped at 2; the increase to a maximum intensity of 2 is achieved in increments that distribute over the full 99-point intensity display scale. For the Revitive sham group, the participant’s familiarization with the device and “Instructions for Use” will be the same as that used for the to active device groups. They will also be advised that the maximum stimulation level may be perceived by some participants but not others and that sensations or feelings often become less noticeable over time. The Revitive sham program runs for 30 min, which is identical to the active devices.

### Criteria for discontinuing or modifying allocated interventions {11b}

N/A—no criteria are specified that warrant discontinuation of the trial intervention.

### Strategies to improve adherence to interventions {11c}

The importance of accumulating 60-min treatment duration per day will be explained to participants at the baseline visit and during device familiarization. Participants will be asked to keep a daily record of treatment sessions undertaken. Participants will be advised that they can contact the investigator at any time with questions relating to study procedures and device use.

### Relevant concomitant care permitted or prohibited during the trial {11d}

During the study, participants will continue with their normal life, activities, medications, and diet with no restrictions.

### Provisions for post-trial care {30}

Any incidental clinical findings identified during study assessments will be reported to the participant, if appropriate, and the participant will be advised to seek medical advice from their general practitioner. Upon completion of the trial, all participants will be offered a new Revitive Medic^©^ Program 1 device, so they can have ongoing access to the trial intervention.

The University of Hertfordshire has provided an agreement to compensate for injury resulting from participation in a sponsored clinical investigation. As such, participants enrolled into the study are covered by indemnity for harm resulting from their participation in the trial.

### Outcomes {12}

The primary endpoint for the study comprises a comparison of change in Canadian Occupational Performance Measure Performance (COPM-P) score from baseline to Week 8 for Revitive Medic^©^ Program 1 versus Revitive sham. COPM-P score is a self-evaluation measure of each participant’s current physical/ occupational performance status.

Secondary endpoints comprise:

- Change in COPM-P from baseline to week 12, comparing Revitive Medic^©^ Program 1 versus Revitive sham.
- Change in COPM-P from baseline to weeks 8 and 12 comparing Revitive^®^ Program 2 and Revitive sham.
- Change in Canadian Occupational Performance Measure Satisfaction (COPM-S) score from baseline to week 8, comparing Revitive Medic^©^ Program 1 versus Revitive sham and Revitive^®^ Program 2 versus Revitive sham. COPM-S score is a self-evaluation measure of each participant’s current satisfaction with their relevant physical/occupational performance.
- Change in leg pain from baseline to weeks 8 and 12, comparing Revitive Medic^©^ Program 1 versus Revitive sham and Revitive^®^ Program 2 versus Revitive sham; participants will rate leg pain using an 11-point numerical rating scale.
- Change in blood flow volume and intensity from baseline during device use, comparing Revitive versus Revitive sham.
- Change in symptom score from baseline to weeks 8 and 12 in terms of overall symptom score and each item (heaviness, tiredness, aching, and cramps), comparing Revitive Medic^©^ Program 1 versus Revitive sham and Revitive^®^ Program 2 versus Revitive sham.

Assessments conducted at the different study visits are presented in Table 1.

**Table 1:** Study assessments in the NMES stimulation trial

	**Telephone assessment (≥ week − 1)**	**Baseline assessment** **(week 0)**	**Treatment period**	**Week 8**	**Week 12**
Eligibility	x	x			
Randomization	x				
Informed consent		x			
Demographics		x			
Medical history	x	x	x	x	x
Medications	x	x	x	x	x
Device issue and familiarization		x			
Device usage		x	x	x	
COPM		x		x	x
Symptom (heaviness, aching, tiredness, and cramps) scores		x		x	x
Leg pain		x		x	x
Doppler ultrasound		x		x^a^	
Adverse events		x	x	x	x

^a^For participants with poor quality Doppler data at week 0

Following the 8-week intervention period, participants will return their device to the research team and will be assessed for Week 8 outcome measures. Participants will then return for a repeat assessment of all outcome measures at Week 12, following a 4-week follow-up period without device use. This post-intervention follow-up period is included to assess the short-term maintenance of the treatment effect.

Safety assessment comprises a formal evaluation of all adverse events (AEs), adverse events of special interest (events with a potential causal association to the use of the study device), and serious adverse events (SAE).

### Participant timeline {13}

**Fig. 1:**
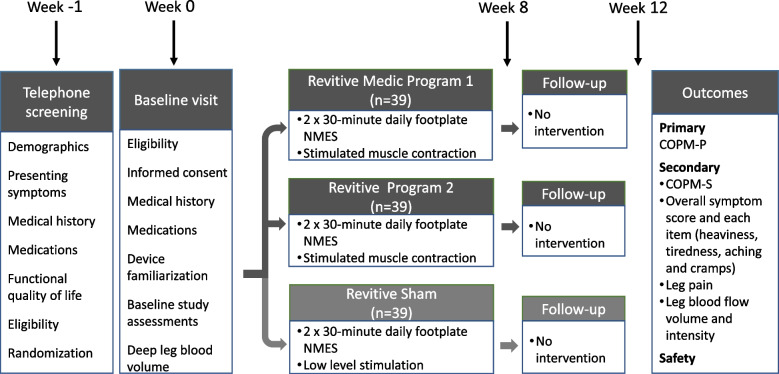
Design of the NMES stimulation trial

Participants will be on-study for 12 weeks, with treatment continued for 8 weeks and study assessments conducted at Weeks 0 (baseline), 8 (post-intervention), and 12 (follow-up) as presented in Fig. 1. Participant duration in the trial will continue for 12 weeks.

Potential participants who express an interest in the study will be contacted by telephone and provided with further information about the study, any questions will be answered and initial screening assessments conducted to inform on eligibility. Once an interested participant is deemed suitable for the study, they will be sent the participant information sheet, randomized, and an appointment will be made to attend the baseline visit; the baseline visit will be arranged for after the participant has had sufficient time to consider the participant information sheet (at least 2 days). Potential participants will be provided with an opportunity to ask questions and request further information about the study at the baseline visit; the participant will also have eligibility criteria checked and provide informed consent. Subsequent procedures will only be initiated once the participant has provided written informed consent.

The participant will undergo baseline assessments and be allocated their study device. Participants will then be instructed as to the correct use of their device and undergo a Doppler assessment of deep leg blood flow volume and intensity both before and during device use. Participants will be sent home with their device to use over the 8-week treatment period.

Participants will return to the study center after 8 weeks (at the end of the treatment period) for study assessments and to return their study device; they will remain on-study for another 4 weeks (follow-up period) during which time they will not use the device. Participants will return to the study center for week 12 assessments, after which they will be considered as having completed the study.

### Sample size {14}

The sample size for the study is 117 participants, with 39 participants in each treatment group.

At the time of protocol development, the anticipated effect of Revitive Medic^©^ Program 1, Revitive^®^ Program 2, and Revitive sham on the primary outcome (COPM-P) was not understood. Multiple publications [27–29] have cited an improvement of 2 points in COPM-P score for an individual participant as a minimally clinically important difference (MCID); a 2-point change in COPM was therefore set as the threshold required for a participant to be considered a responder to the study intervention.

An absolute difference of 30% in the proportion of participants that meet the COPM-P responder definition (improvement of 2 points) between Revitive sham and Revitive Medic^©^ Program 1 or Revitive^®^ Program 2 was considered necessary to demonstrate a clinically meaningful difference for either test device. To control Type I error, a single primary endpoint was chosen namely the difference between Revitive sham and Revitive Medic^©^ Program 1; a comparison of Revitive sham and Revitive^®^ Program 2 is a secondary endpoint. A sequential testing procedure will be employed such that the secondary endpoint can only be formally assessed if the primary endpoint achieves statistical significance (*p* < 0.05).

Basing the sample size calculation on this design, it was determined that 39 participants would be needed in each of the three intervention groups to show an absolute difference of 30% in the proportion of responders between Revitive sham and Revitive Medic^©^ Program 1 or Revitive^®^ Program 2 at 80% power and two-sided 5% significance. For the purposes of the power calculation, the comparison used a Pearson chi-square test at the two-sided significance level (*p* < 0.05).

### Recruitment {15}

Paid print advertising will be used to promote the study to individuals in the community who fit the age requirement and meet study inclusion criteria. Recruitment will also be open to people who hear about the study from friends, relatives, or other sources.

## Assignment of interventions: allocation

### Sequence generation {16a}

Participants will be randomized on study entry to one of three treatment groups in a 1:1:1 ratio using computer-generated blocks; blocks of 9 participants will be used to ensure equal group numbers throughout the recruitment and enrolment period. Study participants will be allocated a Revitive device (identified by a model number which distinguishes Revitive Medic^©^ Program 1, Revitive^®^ Program 2, and Revitive sham) according to the randomization schedule.

### Concealment mechanism {16b}

At the time of recruitment, the researcher will allocate participant numbers sequentially to all participants. Each participant number has a pre-allocated study group as per the concealed randomization table, which the researcher is unaware of at the time of recruitment thus avoiding selection bias. Participants will be blinded to their intervention allocation throughout the trial and informed that they have a one in three chance of being randomized to a particular treatment group. It will not be possible to differentiate active and sham devices, as the packaging and appearance of all devices are identical. The same detailed “Instructions for Use” are given to participants regardless of the device they receive. Description of the sensations that can be experienced while using the device will be the same for all participants (a range of sensations from light tingling and feelings of pins and needles to noticeable muscle twitch and contractions in the feet and legs).

Familiarization and ongoing support with the device will be managed by the investigator, who will provide feedback and cues to the participant that their sensations/feelings in response to using the device are as expected/normal.

### Implementation {16c}

The investigator will generate the allocation sequence and enroll and assign participants to interventions.

## Assignment of interventions: blinding

### Who will be blinded {17a}

This will be a single-blind study with trial participants blinded as to the assignment to their study intervention. Conducting a double-blind study is not feasible, as the therapeutic functioning of the NMES device is dependent on reaching an intensity that results in visible contraction of the calf muscle; as such, the study assessor would be aware of whether the participant used an active or sham device during blood flow measurement.

### Procedure for unblinding if needed {17b}

N/A—this is a single-blind trial with only the participant being unaware of their allocated treatment group, therefore unblinding is not necessary.

## Data collection and management

### Plans for assessment and collection of outcomes {18a}

Outcome and baseline trial data will be collected using a case report form administered by the investigator at each study visit. Images and data from the Doppler ultrasound will be acquired electronically at the baseline visit.

#### Compliance

At the baseline visit, participants will be asked to keep a daily record of treatment sessions undertaken. In the rare event that one or more sessions are missed, participants will be required to keep a written record of those events and report them to the investigator at the week 8 visit. The investigator will document the total number of sessions missed by each participant during the intervention period. Data from participants who miss interventions will be analyzed.

#### Canadian occupational performance measure

The COPM [30] was developed to identify and prioritize participant-specific occupational problems and evaluate changes in these areas using a semi-structured interview; the individual is encouraged to identify activities that he or she wants, needs, or is expected to do but cannot, or those in which the individual is not satisfied with their current performance. The COPM is a generic measure meaning it can be used in all populations, as long as participants are able to reflect on their lives and activities and communicate on these aspects.

The COPM has been assessed as a validated tool that provides novel information when used to assess change in score over time for an individual participant [31–33].

COPM is considered a valid, reliable, clinically useful, and responsive outcome measure and has been widely used to assess change in interventional studies in community populations including the elderly [28, 34, 35].

#### Pain and symptom scores

Pain and symptom scores will be assessed at weeks 0, 8, and 12 with participants asked to indicate pain and symptoms (heaviness, tiredness, aching, cramps) they experience and rank them on a 0 to 10 numerical rating scale.

#### Deep leg blood flow volume and flow intensity

Deep leg blood volume will be assessed using Doppler ultrasound before and during the use of the Revitive device [36]; the IsoRocker on the Revitive device will be disabled during Doppler ultrasound, to minimize movement during assessment. Blood flow will be measured at the medial aspect of the ankle between the medial malleolus and the Achilles tendon, using the appropriate arterial ultrasound probe and pre-set volume flow algorithms on a duplex ultrasound machine. Two sets of recordings will be taken, pre-stimulation and 30 s into Waveform 5. Five seconds worth of data will be recorded at each time. The recording will then be broken down into individual frames, saved onto the scanner, and analyzed mathematically by MATLAB [36]. MATLAB analysis of frames determines peak flow volume and the peak intensity of flow within those 5 s. If baseline visit data are of insufficient quality due to noise, Doppler ultrasound can be repeated at week 8.

### Plans to promote participant retention and complete follow-up {18b}

Participants will be advised at their baseline assessment visit that telephone/email/text/social media support is available and they will be encouraged to contact the trial investigator to discuss any questions or difficulties they have.

Participants who discontinue the trial early will be asked if they agree to completing follow-up and having their data included in the final analysis.

### Data management {19}

Study data will be transferred, by the investigator, from the paper case report form into an electronic database on completion of subject participation. Hard-copy data will be stored in secure locked storage at the study site; electronic data will be stored at the study site using sponsor data security systems. Significant data errors (range checks) will be monitored for during data analysis. Data analyses will be conducted and reported in accordance with Consolidated Standards Of Reporting Trial guidelines for non-drug trials.

### Confidentiality {27}

All data will be handled in accordance with the UK Data Protection Act 2018. Case report forms do not bear the participant’s name or other identifiable personal data. Participants will be sequentially assigned a trial identification number upon enrolment into the study, and the study site will maintain a master participant identification log.

All individual participant information will be de-identified during the reporting of data and resulting publications or presentations, to fully protect participant confidentiality. Participants will be informed that information or reports from the study will be prepared and submitted for publication. Participant information will normally be presented as group data. If necessary, information obtained from specific individuals may be presented but names will not be used. Participants will only be identified in such publications by their identification number and possibly their age and gender.

### Plans for collection, laboratory evaluation, and storage of biological specimens for genetic or molecular analysis in this trial/future use {33}

N/A—no biological specimens will be collected.

## Statistical methods

### Statistical methods for primary and secondary outcomes {20a}

Analysis of covariance (ANCOVA) will be used to compare Revitive Medic^©^ Program 1 versus Revitive sham and Revitive^®^ Program 2 versus Revitive sham at weeks 8 and 12, with change from baseline as the dependent variable, and treatment group and baseline value as independent variables. Least square means for each group, treatment differences, 95% confidence intervals (CIs) and *p*-values will be calculated. Model assumptions will be checked and, if departures from normality are evident, a Wilcoxon rank sum test and associated 95% CI using the Hodges Lehmann estimator will be used to compare groups.

Logistic regression with baseline value as a covariate and treatment group as a classification variable will be used to compare the proportion of responders in each test group versus the sham group. Treatment effect will be estimated as an odds ratio (test/sham), with 95% CIs and associated *p*-value. An odds ratio of > 1 indicates a better outcome in the test group.

For all parameters at each time-point, only participants with both a baseline and the corresponding post-treatment assessment will be included in the calculation of change from baseline.

Any subgroup analysis based on factors such as, for example, age or co-morbidities in a study of this size (< 40 per group) is going to have limited power to show differences within these subgroups. Therefore, any such analysis will be of limited value. However, further exploratory analyses, investigating the effects of other covariates (such as age and BMI), may be undertaken for the study outcomes and provided as supportive information.

#### Canadian occupational performance measure performance score

The best possible score for COPM-P is 10 points. Any participant with a score of 10 at baseline will be excluded from the modified intent-to-treat (mITT) population, since the condition of interest is absent in these participants. Gains of at least 2 points in COPM are considered clinically important (MCID; 27–30). Using this threshold, participants will be classified as either a responder (change in COPM-P of ≥ 2) or non-responder (change in COPM-P of < 2) at weeks 8 and 12. The proportion of responders at each time point will be summarized by treatment group. Responder analysis will be performed for the intent-to-treat (ITT), mITT, per protocol (PP) populations, and also the subset of participants with a baseline score of ≤ 8 (since participants with a baseline score of > 8 cannot meet the MCID).

For ANCOVA and logistic regression, a hierarchical testing procedure will be used to maintain the Type I error rate at 5%. Firstly, the statistical significance of Revitive Medic^©^ Program 1 versus Revitive sham will be calculated, and if the *p*-value is ≤ 0.05 testing will proceed to Revitive^®^ Program 2 versus Revitive sham (also at the 5% significance level). If Revitive Medic^©^ Program 1 versus Revitive sham has a *p*-value of > 0.05, then the difference between Revitive^®^ Program 2 and Revitive sham will be considered non-significant but a *p*-value will be presented for descriptive purposes.

#### Canadian occupational performance measure satisfaction score.

COPM-S data will be summarized and analyzed in the same way as COPM-P. The best possible score for COPM-S is 10 points and the MCID for COPM-S is 2 points [27–29]. COPM-S analyses will be performed for the same study populations as defined for COPM-P.

#### Leg pain

Any participant who has no pain (pain score of 0) at baseline will be excluded from the mITT analysis since the condition of interest is absent. A change in pain score of 2 (measured on an 11-point numerical rating scale) is recognized as meaningful [37–39]; participants will, therefore, be classified as responders if their pain score improves by at least 2 points from baseline. The proportion of responders at Weeks 8 and 12 will be summarized by treatment group. The hierarchical test procedure will be used to evaluate the effectiveness of Revitive Medic^©^ Program 1 and Revitive^®^ Program 2. Responder analysis will be performed for the ITT, mITT, PP, and also the subset of participants with a baseline pain score of ≥ 2 (since participants with a baseline score of < 2 cannot meet the criteria for a clinically meaningful change).

#### Symptom score

Symptoms will be assessed at weeks 0, 8, and 12. A total symptom score will be calculated as: (the number of symptom days multiplied by the average intensity, summed across all 4 symptom domains) divided by 7. Total score can range from 0 (best outcome) to 40 (worst outcome). A score of 0 indicates that the symptom was not present but will be included in the calculation of total symptom score, since this provides valuable data on symptom totality. Any participant with a total score of zero at baseline will be excluded from the mITT analysis as the condition of interest is absent.

A domain score for each item will be calculated as: (the number of days multiplied by the average intensity) divided by 7. Each domain score can range from 0 (best outcome) to 10 (worst outcome). For calculation of individual domain scores, only symptoms present at baseline will contribute to the baseline and post-baseline symptom scores for the mITT analysis. Change to Weeks 8 and 12 will then indicate the evolution of symptoms that were present at baseline.

#### Deep leg blood flow

Blood volume and blood intensity will be measured using Doppler ultrasound before and during NMES. If week 0 data are deemed erroneous due to noise in Doppler images, the assessment can be repeated at week 8; the data considered of the best quality with minimal noise will be recorded. Since blood flow is only measured under a single waveform, Revitive Medic^©^ Program 1 and Revitive^®^ Program 2 are not distinguishable; for this endpoint, data for Revitive Medic^©^ Program 1 and Revitive^®^ Program 2 groups will be combined and summarized as a single group.

### Interim analyses {21b}

To inform on response rates and provide baseline data for sample size calculation, a pilot study was conducted with the first 10 participants from each of the three groups (30 participants in total). Based on the pilot study, the responder rate was calculated for the Revitive sham, and an absolute risk difference was defined for determining the responder rate for Revitive Medic^©^ Program 1 and Revitive^®^ Program 2 needed to show treatment benefit. The difference in responder rates was then used to calculate the total sample size for the study. Data from the 30 participants from the pilot study will be included in the final analysis, as they followed the same protocol as subsequent participants. No hypothesis testing for stopping for futility or efficacy was conducted at the end of the pilot, and so the potential for inflation of Type I or Type II errors is considered negligible.

### Methods for additional analyses (e.g., subgroup analyses) {20b}

N/A—no additional analyses are planned.

### Methods in analysis to handle protocol non-adherence and any statistical methods to handle missing data {20c}

Data from this trial will be summarized and analyzed for the ITT, mITT, and PP populations; analysis of the mITT and PP populations will be considered secondary.

- The ITT population will include all enrolled and eligible participants who were randomized and used their assigned device at least once. The ITT population will be used to summarize demographic, device compliance, and all efficacy parameters.
- The mITT will include all enrolled and eligible participants who were randomized and used their assigned device at least once, and for whom the condition being assessed was present at baseline; a separate mITT population will be defined for COPM, symptom score, and leg pain, as these depend on the baseline score for each parameter. In cases where ITT and mITT populations are identical, only the ITT analysis will be presented.
- The PP population will include participants from the ITT population who were compliant with using their assigned device, defined as missing no more than 28 treatment sessions (minimum 75% compliance). Participants unable to confirm how many treatment sessions they missed will be excluded from the PP population. The PP population will be used to summarize COPM, symptom score, leg pain, and blood volume and intensity data.

Participants who discontinue the study will have data collected up to the point of discontinuation. For participants not assessed at week 8 or 12, the value will be left as missing for calculation and presentation of summary statistics. Participants who do not return their study device at the week 8 visit will be considered as having discontinued the study at this time; this ensures that the potential for continued use of the device after week 8 does not compromise study findings.

For statistical analysis, multiple imputation will be used to handle missing data. It will be assumed that data are missing at random. If the pattern of missing data is non-monotone, then partial imputation will initially be carried out (just enough to get the monotone missing data pattern) using the Markov Chain Monte Carlo method. Once the data exhibit a monotone missing data pattern, the monotone regression method will be used to impute the remaining missing data. The regression model will include terms for treatment and the observed values at visits prior to the missing value. For each endpoint, 20 imputed datasets will be created and analyzed, and the results will be pooled using the MIANALYZE procedure. Sensitivity analysis will be performed whereby missing data will be replaced by the value recorded at baseline (baseline observation carried forward).

For COPM-P responder analyses, participants who do not have an assessment for a visit will be categorized as responders or non-responders according to the value obtained via the multiple imputation; for sensitivity analysis, they will be classified a non-responder (non-responder imputation) at that visit.

### Plans to give access to the full protocol, participant-level data and statistical code {31c}

Study staff involved in the collection and analysis of data will have access to the trial data set. Access to the final cleaned dataset will be provided to the trial statistician for the purpose of carrying out the planned analyses. Ongoing access to the data will only be available to the study team for the purpose of carrying out related analyses.

## Oversight and monitoring

### Composition of the coordinating center and trial steering committee {5d}

N/A—this study will be conducted by the study investigator, who is responsible for ensuring that the trial is conducted according to the Medical Research Council Guidelines for Good Clinical Practice in Clinical Trials.

### Composition of the data monitoring committee, its role and reporting structure {21a}

N/A—the Revitive NMES device is a commercially available, CE-marked, Class II device with extensive use in the community setting, without safety signals occurring. As a result, the use of a data monitoring committee was not considered necessary.

### Adverse event reporting and harms {22}

The occurrence of AEs will be recorded throughout the study. Participants will be asked about the occurrence of such events at study visits. Adverse events of special interest will be identified as events with a potential causal association to the use of the study device. Any SAEs will be investigated by an independent medical monitor and reported to the ethics committee. A safety board will be convened if deemed necessary, e.g., where a trend in incidents or events is noted.

The Revitive NMES device is a commercially available, CE-marked, Class II device with extensive use in the community setting. As such the risk of SAEs is expected to be rare. Standard risk assessment was conducted for both the investigational device and the clinical investigation process and identified that all risks associated with the study were adequately controlled.

### Frequency and plans for auditing trial conduct {23}

Sponsor audits will follow University of Hertfordshire procedures for internal studies, whereby annual monitoring is initiated once the study has received ethics committee approval.

### Plans for communicating important protocol amendments to relevant parties (e.g., trial participants, ethical committees) {25}

The protocol, consent form, and materials given to prospective participants will be reviewed and approved by the ethics committee responsible for oversight of the study before participants are enrolled. In addition, all local and site-specific research and clinical governance approvals will be obtained prior to participant enrolment.

Subsequent protocol modifications will be approved by the ethics committee and the sponsor and investigator will be responsible for communicating changes to relevant parties.

### Dissemination plans {31a}

Study results will be published in an international, high-impact, peer-reviewed journal and made available to the research community. The study protocol and/or results may be used in national and/or international conference presentations.

Feedback will be provided to trial participants, which will include a summary of outcomes and information as to which intervention group they were assigned to. This feedback will be prepared in a brief report and participants will have the opportunity to ask questions.

## Discussion

NMES is an established therapy that has been widely used for many decades to improve circulation in the legs [20–22]. However, sham-controlled studies of the use of such devices in an elderly, ambulant, and apparently healthy population are lacking. This study aims to provide data informing on the applicability of the use of NMES for the management of leg symptoms that are likely to be indicative of poor blood flow in an elderly population.

Lower leg symptoms such as leg aching/pain, heaviness, tiredness, and cramps are common in older adults and impact considerably on physical functioning and quality of life. These symptoms are often associated with poor blood flow in the lower limbs and can arise due to prolonged inactivity, a sedentary lifestyle and/or be associated with undiagnosed peripheral artery disease [1, 4, 6, 10]. A “vicious cycle” model describes the progressive nature of such symptoms; pain and discomfort limit physical activity, and these consequential limitations in activity further worsen lower leg symptoms [3].

Foot NMES has been proven to increase blood circulation in the legs during use and may help relieve symptoms such as pain, swelling, heaviness, cramps, and tiredness. When NMES is applied to the plantar surface it indirectly induces contraction of the calf muscle, activating the musculo-venous pump and improving circulation, and thereby promoting movement of excess fluid away from the legs [19]; contraction of the muscles of the lower leg and feet also improves muscle tone.

The results of this study should inform on the use of NMES in community-dwelling adults aged > 65 years who have leg pain and symptoms. NMES devices are intended to improve circulation in the legs and though studies have confirmed a beneficial effect in healthy individuals, the literature generally describes use of the intervention in younger assessment groups (mean 29 to 33 years; 16, 23). Specifically, the use of the Revitive device in an elderly, healthy population with leg pain and symptoms generally considered consistent with aging has not previously been investigated.

This study has various strengths and some weaknesses. To our knowledge, this is the first study to investigate the effect of foot NMES on the physical function and leg symptoms among community-dwelling older adults with potential underlying issues with leg circulation. The study features a sham control and is adequately statistically powered. The primary outcome measure in the study is “patient-centered” and evaluates physical function at the level that is important to the participants. However, one inherent limitation of the study is that the assessor is not blinded to the study group allocation. The presence of visible muscle contraction with the active NMES devices makes it impossible to blind the assessor.

## Trial status

The current protocol version is 4.0 dated 21 June 2022. Recruitment into the study started on 01 July 2019 and completed on 30 June 2022. The estimated study completion date is 31 October 2022. It was not possible to submit this manuscript before recruitment completed, as recruitment and manuscript preparation were delayed by the COVID-19 pandemic; trending recruitment indicated completion by the end of 2022. However; subsequent to COVID, recruitment proceeded much faster and outpaced the preparation of this manuscript.

## Data Availability

Upon completion of the trial, Actegy Limited, the device manufacturer and funder of the study, will be transferred the trial data. The data transferred to Actegy Limited will not include source documents or medical records and will be fully anonymized.

## Abbreviations

AE: Adverse event
ANCOVA: Analysis of covariance
CIs: Confidence intervals
COPM-P: Canadian Occupational Performance Measure Performance
COPM-S: Canadian Occupational Performance Measure Satisfaction
ITT: Intent-to-treat
MCID: Minimally clinically important difference
mITT: Modified intent-to-treat
NMES: Neuromuscular electrical stimulation/stimulator
PP: Per protocol
SAE: Serious adverse event
